# Effect of Wnt10a/β-catenin signaling pathway on promoting the repair of different types of dentin-pulp injury

**DOI:** 10.1007/s11626-023-00785-z

**Published:** 2023-09-12

**Authors:** Yue Li, Meiying Wu, Xinyu Xing, Xingxing Li, Congchong Shi

**Affiliations:** 1 Department of Orthodontics, Kunming Medical University School and Hospital of Stomatology, Kunming, 650106 China; 2 Yunnan Key Laboratory of Stomatology, Kunming, 650106 China; 3 Department of Prosthodontics, Kunming Medical University School and Hospital of Stomatology, Kunming, 650106 China

**Keywords:** DPSCs, Dentin-pulp complex injury, Wnt10a/β-catenin signaling pathway

## Abstract

How to repair dentin-pulp injury effectively has always been a clinical problem, and the comparative study of repair process between different injuries is unknown. Dental pulp stem cells (DPSCs) often are selected as seed cells for the study of dentin-pulp injury repair due to excellent advantages in odontogenesis and pulp differentiation. Although many previous researches have indicated that the Wnt protein and Wnt/β-catenin signaling pathway were crucial for dental growth, development, and injury repair, the specific mechanism remained unknown. In this study, different dentine-pulp injury models of adult mice were established successfully by abrasion and cutting methods. The gross morphology and micro-CT were used to observe the repair of injured mice incisor in different groups. We found that the repair time of each group was different. The repair time of the cutting group was longer than the abrasion group and the qRT-PCR detection showed that the expression of DSPP in the cutting group was higher than that in the abrasion group, but there was no significant difference in proliferation among the groups. In vivo and cell experiments showed that activation of Wnt/β-catenin signaling pathway can promote the proliferation and odontoblast differentiation of DPSCs. In addition, by using RNAscope staining, we observed that Wnt10a was mainly expressed in the proliferative region and partially expressed in the odontoblast region. The Western blotting results showed that in the early stage of repair, the expression of Wnt10a increased with the extension of days after injury in both abrasion and cutting group and the increase of Wnt10a was tested obviously on the 5th day after injury. But on the 7th day after injury, the expression of Wnt10a was still obvious in the cutting group, while the expression of Wnt10a was significantly reduced in the abrasion group, which was close to the control group. It is suggested that Wnt10a acts as a repair-related protein and has an important role in tooth injury repair. Wnt10a was activated by R-spondin and LiCl, and Wnt10a-siRNA DPSCs were constructed to inhibit Wnt10a. The results showed that Wnt10a/β-catenin signaling pathway promoted the proliferation and odontoblast differentiation of DPSCs. It plays a crucial role in the repair process of different injuries. This study enriched the mechanisms of Wnt10a /β-catenin signaling pathways in different types of dentin-pulp injury repair, which could provide experimental evidences for the target gene screening and also give some new ideas for the subsequent research on the molecular mechanisms of tooth regeneration.

**Graphical Abstract:**

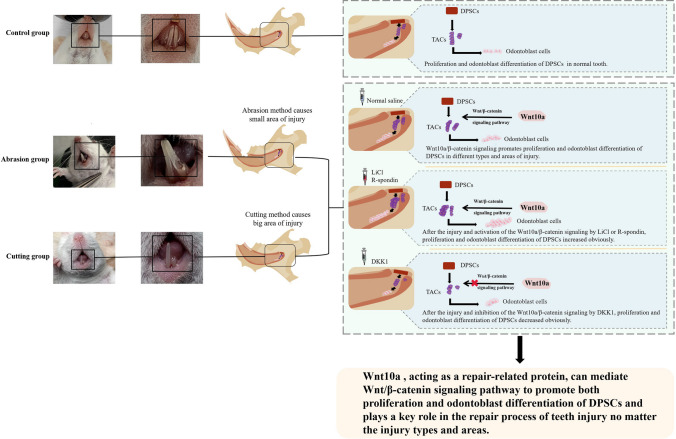

## Introduction

Dentin-pulp injury caused by dental caries, trauma, or iatrogenic causes will not only lead to various diseases of dental and periodontal tissues, such as pulp infection, tooth loosening, and even loss, but also affect facial beauty and masticatory function, as well as the mental health of patients (Anthony et al. 2016). It has been reported that, there are about 2 billion people and 514 million children suffering from dental caries in permanent teeth and deciduous teeth respectively worldwide. Also, the incidence of dental injury is generally high around the world, about 15.2% in deciduous teeth and 22.7% in permanent teeth (Chai and Slavkin 2003). Therefore, how to repair dental damage more efficiently has become one of the research hotspots.

To study the process and mechanism of dental injury repair more intuitively and accurately, the establishment of animal injury models are the important parts. At present, abrasion and cutting methods are often used. However, there are no specific comparative studies on the repair and signaling pathway regulation among different injury models.

Dental pulp stem cells (DPSCs) have a broad range of applications in regenerative medicine and stem cell research, because they have high differentiation potential, have a wide source, and are available widely, have no ethical controversy, and are easy to preserve and culture. Besides, they are rarely damaged to the donor and recipient. They can significantly reduce the cost of stem cell therapy and facilitate the development of research. Moreover, DPSCs have low immunogenicity, have excellent safety, and are less prone to tumor formation, which is beneficial to injury repair. At the same time, DPSCs are considered to be a key factor in the process of dentin-pulp injury repair. They achieve this goal primarily by migrating to the site of injury and then differentiating into odontoblast-like cells that produce repaired dentin (Tsutsui 2020).

The incisors of mice continue to grow throughout their lives, including the constant renewal of ameloblasts and odontoblasts (Sønstevold et al. 2015). This provides an attractive model system for studying stem cells which facilitate homeostasis, regeneration, and repair of tissues (About 2013; Yu and Klein 2020).

Several adult tissues include skin and teeth, which rely on Wnt signaling for almost all aspects of embryogenesis, organ development, tissue regeneration, and homeostasis self-renewal; a variety of Wnt proteins play an important role in regulating cell behavior, proliferation, and fate through the Wnt signaling pathway. The Wnt/β-catenin signaling pathway significantly affects the proliferation of DPSCs and the development of teeth differentiation. Wnt10a, a member of this family, is closely related to this function (Doolan et al. 2021). Previous studies about Wnt10a mostly focused on the formation of root bifurcation and congenital tooth loss (Park et al. 2019; Yu et al. 2020). Others found that Wnt10a is related to tooth size, hypoplasia, and pulp loss. However, it is not clear how the Wnt/β-catenin signaling pathway mediated by Wnt10a accurately regulates the proliferation of DPSCs and the differentiation of odontoblast cells in the whole process of the repair of different types of dentin-pulp injury, and then promotes the repair of incisor injury.

In this study, mice models of dentin-pulp injury were successfully established by two different ways to simulate common clinical injuries and to investigate the effects of Wnt10a and Wnt/β-catenin signaling pathways on the repair of dentin-pulp injury. Our results demonstrated that Wnt10a/β-catenin signaling pathway has beneficial regulatory effects on the proliferation and odontoblast differentiation of DPSCs and the process of incisor repair. It provides new insights and ideas for the mechanism of teeth repair.

## Materials and methods

### Experimental animals

Seventy SPF-grade healthy 8-wk-old female Kunming mice, weighing 28–30 g, were purchased from the Department of Laboratory Animals of Kunming Medical University. The experimental program has been approved by the Animal Ethics Committee of Kunming Medical University (approval number: KMMU2020292).

### Reagents

The reagents used in this study were anhydrous lithium chloride (C8381, Solarbio, Beijing, China) prepared as a solution of 0.75 g/L as required, DKK1 recombinant protein (H00022943-P01, Abnova) prepared at a concentration of 1 µg/ml, R-spondin protein (ZN2748, Biorad, Hercules, CA) prepared in 1 µg/ml concentration, BCA protein quantification kit (P0009, Beyotime, Shanghai, China), Trizol (15,596–026, Ambion, Naugatuck, CT), RNA Reverse Transcription Kit (G3337-50, Ambion), Real-Time Fluorescence RT-PCR Reagent (G3325-15, Service), Ki67 Antibody (ab15580, Abcam), Chondrogenic Induction Kit (MUCMX-90041, Saiye Biotech Ltd. Guangdong Sheng, China), EDU (C0071S, Beyotime, Shanghai, China), and Lipofectamine RNAiMAX (13,778,030, Thermo Fisher, Waltham, MA).

### Instruments

The instruments employed in this study were the real-time fluorescence quantitative PCR instrument (788BR08065, BIO.RAD), the Optical Microscope (LEICA DM750 ZEISS, Wetzlar Germany), and the Protein Electrophoresis Instrument (165–8033, BIO-RAD), NEMO® NMC-100 micro-CT system (PINGSENG Healthcare Inc., Kunshan, China).

### Mice incisor injury model assay

Experimental animal groups: Seventy Kunming mice were randomly divided into 7 groups, each group consisted of 10 mice. The seven groups are the control group, the abrasion model group, the abrasion LiCl group, the abrasion DKK1 group, the cutting model group, the cutting LiCl group, and the cutting DKK1 group. The abrasion model group, the abrasion LiCl group, and the abrasion DKK1 group used the method of abrasing an incisor. The cutting model group, the cutting LiCl group, and the cutting DKK1 group used the method of cutting an incisor. No treatment was done in the control group.

**Figure. 1:**
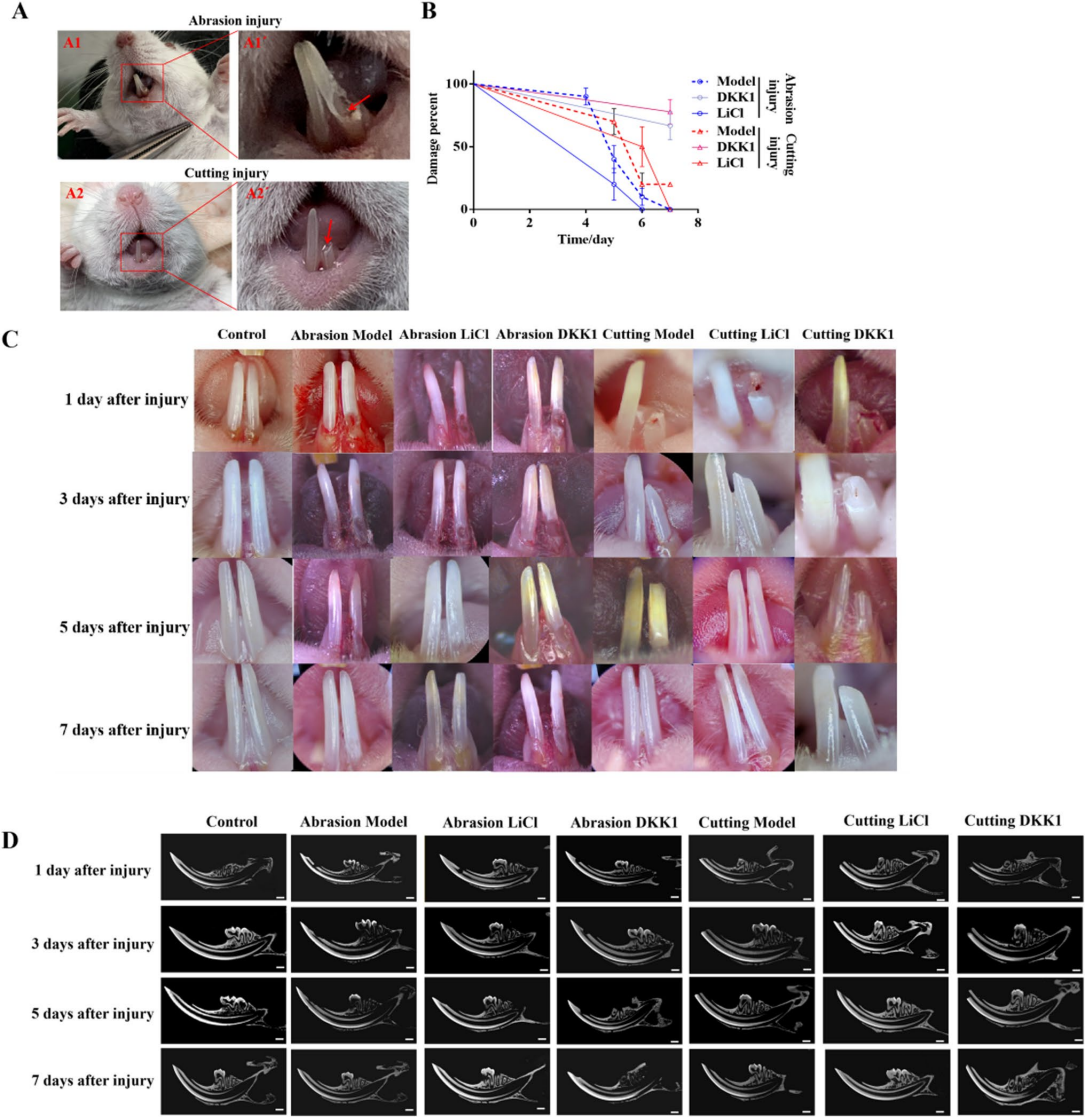
Observation and analysis of different injury models of mice incisor teeth. After successful establishment of the mice model and continuous injection of drugs for 7 d, observing the repair of dentin-pulp injury in the abrasion model, abrasion LiCl, abrasion DKK1, cutting model, cutting LiCl and cutting DKK1 groups at 1, 3, 5, and 7 d after injury. (*A*) The dentin-pulp injury model of abrasion and cutting methods was successfully established. (A1, A1') The dentin-pulp injury model was established in the abrasion method, and the *red* *arrow* indicated the red pulp point which was visible. (A2, A2') The dentin-pulp injury model was established in a cutting method, and the red pulp point which was indicated by the *red arrow* was visible. (*B*) The repair time and percentage of dentin-pulp injury in each group after the successful establishment of the mice model and continuous injection of drugs for 7 d. Percentage of injury = (number of mice with unrepaired incisor teeth in each group/10) × 100%. (*C*) Morphological observation. With the increase of time after injury, the defect area of the incisor tissue of mice in each group was gradually reduced and repaired to the same smoothness and length as the normal teeth on the opposite side. (*D*) Micro-CT observation. With the increase of time after injury, the defect area of the incisor tissue of mice in each group gradually decreased, and the defect at the dentin layer was gradually formed until it was completely repaired. The internal structure of repaired teeth were consistent with the control group. *Scale bar*, 1 mm.

The abrasion method: We used a dental slow-speed handpiece (EX-203C Set, NSK) connected to the planting machine power system (ICT motor Serial NO.B02DED1021, Dentium) and coupled with a 1-mm diameter tungsten steel slow bending ball drill (RA2, Dentsply, Sirona, Charlotte, NC) to abrade the root of one side incisor. During the abrasion process, the ball drill was stopped when the drill bit could fully enter the tooth tissue and the red pulp piercing point was visible to the naked eye (Appendix Fig. 6(*A*)). Cooling was done with saline while grinding. The diameter of the defect was 1.0–1.5 mm and the depth was 1.0 mm. The other incisor was used as a control for tissue repair of the injured side (Fig. 1(A1, A1')).

 The cutting method: One side incisor was cut directly with ophthalmic scissors until the broken end of the incisor was horizontal with the lingual gingiva (Appendix Fig. 6(*B*)). The red pulp penetration point was visible to the naked eye and the other incisor was used as a control for tissue repair of the injured side (Fig. 1(A2, A2')).

 Drug treatment: For the periodontal ligament injection method, a needle was inserted into the labial gingival groove of the normal incisor in the control group and the damaged incisor in the injured group. The inclined surface of the needle was close to the tooth surface and the needle direction was at an angle of 45° to the long axis of the tooth. Then the periodontal membrane was penetrated slowly and the drug was injected (Appendix Fig. 7). The abrasion LiCl group and the cutting LiCl group were given LiCl solution (0.75 g/L) at a dose of 0.2 ml/kg by local injection into the periodontal ligament, once every 2 days. The mice in the abrasion DKK1 group and the cutting DKK1 group were also injected with DKK1 protein at a dose of 0.2 μg/kg into the periodontal ligament, once every 2 days. The abrasion model group and the cutting model group were injected with an equal volume of saline, once every 2 d. The treatment lasted until the 7th day after the model was constructed successfully. The control group did not receive any treatment. The grouping of animals in this study and the drug treatment method, drug treatment time, and drug dose are shown in Table 1.

**Table 1.** Grouping of experimental animals and drug treatment

Animal grouping	Drug treatment method	Drug treatment time	Drug dose
Control	No receive treatment		
Abrasion model	Abrasion method + saline	Drug treatment at 1, 3, 5, and 7 days after injury	Saline at a dose of 0.2 mL/kg
Abrasion LiCl	Abrasion method + LiCl		LiCl solution (0.75 g/L) at a dose of 0.2 ml/kg
Abrasion DKK1	Abrasion method + DKK1		DKK1 protein (1ug/ml) at a dose of 0.2 μg/kg
Cutting model	Cutting method + saline		Saline at a dose of 0.2 mL/kg
Cutting LiCl	Cutting method + LiCl		LiCl solution (0.75 g/L) at a dose of 0.2 ml/kg
Cutting DKK1	Cutting method + DKK1		DKK1 protein (1ug/ml) at a dose of 0.2 μg/kg

### qRT-PCR analysis

Five mice were sacrificed by excessive carbon dioxide and cervical dislocation. Then we put them in 75% alcohol to disinfect for 5 min. The mandibles were isolated and the soft tissues attached to the mandibles were removed and the mandibles were rinsed with phosphate-buffered saline (PBS) containing 5% diamantine for 2–3 times until there was no obvious blood clot on the tooth surface. Part of the tooth tissue of the tooth cusp and root tip was cut until the pulp point was visible and exposed. Then the pulp in the tooth was flushed out with MEM base medium through a 1-ml syringe and the pulp in the pulp cavity was removed, then rinsing again. The rinse solution was collected and centrifuged at 1000 rpm/5 min, and the precipitate after centrifugation was pulp tissue (Appendix Fig. 8). We extracted an appropriate amount of dental pulp tissue from all groups of mice, added trizol lysis solution to fully lyse, and added chloroform to extract RNA at the ratio of 200ul chloroform per 1 ml of lysis solution. Next, we used the centrifuge at 12,000 × *g*, for 15 min at 4℃, took the supernatant, added 0.5 times the volume of isopropanol to precipitate RNA, used the centrifuge at 12,000 × *g* for 10 min at 4℃, and discarded the supernatant. In the next step, we added 0.5 ml of 75% ethanol to the precipitate for suspending and washing, used the centrifuge at 7500 rpm for 5 min at 4℃, discarded the supernatant, and blotted the mouth of the tube with absorbent paper. Then we opened the lid on the ultra-clean workbench and dried it for 5 min in the air. After that, we added 30–50 µl of DEPC-treated ddH_2_O to dissolve the precipitate according to the amount of precipitate, which was total RNA. Referring to the instructions of the SureScriptTM First-Strand cDNA Synthesis Kit, we took 1 µg of total RNA, added 1 µl of SureScriptRTase Mix (20 ×) and 4 µl of SureScript RT Reaction Buffer (5 ×), and replenished water to 20 µl and under general conditions (25℃ for 5 min, 42℃ for 45 min, 85℃ for 5 min, and 4℃ hold) for reverse transcription on a common PCR machine (4,359,659, Applied Biosystems 2720 Thermal Cycler, USA) to synthesize the first-strand cDNA. Then we used the BlazeTaq SYBR Green qPCR Mix 2.0 to detect the expression of Axin2 and β-catenin on a CFX96 real-time quantitative PCR instrument (Bio-Rad) by using the Sybrgreen method with cDNA as a template and GAPDH as an internal reference. PCR amplification reaction conditions were as follows: 95℃ preheating for 10 min, 95℃ for the 10 s, 60℃ for the 20 s, 72℃ for the 30 s, for 40 cycles, collected and recorded the fluorescence, read the Ct value, and used the 2^–△△Ct^ method to calculate the gene relative expression. All the primer sequences required for this study are shown in Table 2.

**Table 2.** The primer information table

Primers	Sense primer (5′ → 3′)	Anti-sense primer (5′ → 3′)
Wnt4 Wnt5 Wnt6 Wnt7 Wnt10a	GGCTCCTGCGAGGTAAAG CAACTGGCAGGACTTTCTCAA AGGCGGAGCCGGAAGTA CCTTGTTGCGCTTGTTCTCC GGCAGATGGAGGTGTGTGTG	TGTCCTGCTCACAGAAGTCC CATCTCCGATGCCGGAACT TCCAGGAGTGCCCAGAAGG GGCGGGGCAATCCACATAG AGGAAGTATGGCCGGGTGTT
DSPP	CTCTGTGGCTGTGCCTCTTCTA	CAGTGTTCCCCTGTTCGTTTAC
GAPDH	TTGGTCAGGCAAGGGAA	CCCATCACCATCTTCCAGG

### Immunofluorescence staining - Ki67 immunofluorescence staining

 Two mice in each group were sacrificed, the mandibles were isolated, and the soft tissues attached to the mandibles were removed. The mice were placed in 4% PFA at 4°C overnight, and 10% EDTA (PH 7.4) was decalcified for about 4 wk. Dehydration was carried out in ethanol of different concentrations of 50%, 70%, 80%, 90%, and 100% respectively, and the dehydration time of each concentration was 1 h. Transparent with xylene, 1 h each time, repeated 3 times, and then xylene and liquid paraffin were pressed 1:1. Mix, put the sample into the mixture, and put it in a 58°C temperature box for 2 h, then put the liquid paraffin and put it in a 58°C temperature box for 2 h, repeat twice. Paraffin embedding was performed, and after the paraffin was completely hardened at room temperature, paraffin sections were prepared with a thickness of 9 µm, and section information was marked in detail (Appendix Fig. 9). The paraffin sections were dried at room temperature for 30–50 min. Then we washed the sections with 1 × PBS for 5 min and repeated 3 times. We dissolved TritonTMX-100 in PBS at 1:100 for cell membrane perforations, applied at room temperature for 10 min, then washed with PBST for 5 min and repeated 3 times. Next, the cells were sealed with blocking buffer for 1 h at room temperature and primary antibody Ki67 (1:100) and secondary antibody (1:200) were added sequentially. Subsequently, nuclear staining was performed by using DAPI (1:100), incubated for 5 min at room temperature. We protected them from light, washed them for 5 min in PBST, and repeated them 3 times. Finally, fluorescence microscopy was used for image acquisition and analyzed the changes in Ki67-positive cells by Image J image software.

### Expressions of Wnt10a and Axin2 in normal mouse incisors were observed by RNAscope in situ hybridization

Three rats in the 8-week-old normal group were sacrificed, the mandible was isolated, and the soft tissue attached to the mandible was removed. The samples were placed in 10% formalin solution overnight at 4°C and decalcified with 10%EDTA (pH 7.4) for about 4 weeks. Gradient dehydration was carried out with the mixture of 15%, 30% sucrose water, and 60% sucrose water and O.C.T (1:1) successively. The paraffin-embedded tissue blocks were placed on dry ice. After the embedded tissue blocks were hard set, paraffin sections with thickness of 9 μm were taken by sectioning mechanism.

RNAscope in situ hybridization steps are as follows: (1) section drying and cleaning—the section was placed at room temperature or 40℃ temperature box drying for 1 h, 1 × PBS cleaning, 3 min each time, repeated 3 times; (2) antigen repair—the 10 × antigen repair solution in the kit is diluted with dH_2_O at 1:10, heated to 80–85℃, and the sections are put into the antigen repair solution for about 3–5 min. The repair time varies according to different probes; then wash in dH_2_O, 2 min each time, repeat twice; dehydration with 100% ethanol, 2 min each time, repeat twice; remove the slices and place them in room temperature to dry thoroughly; (3) penetration—add the ProteasePlus in the RNAscope Pretreat Reagents H_2_O_2_ and ProteasePlus kit to the slice, acting at room temperature for about 15 min, dH_2_O cleaning, 2 min each time, repeat 2 times; (4) probe hybridization—RNAscope patented Z-type probe Dspp was hybridized with target RNA designed for target gene. The probe should be preheated in a water bath at 40℃, and then react with the tissue. The probe should be incubated in the bath at 40℃ for 2 h. The probe should be washed twice with 2 min each time. (5) Signal amplification: AMP1 in RNAscope 2.5 HD Reagent Kit-Red assay Kit was added and incubated at 40℃ for half an hour, then washed with wash buffer twice, 2 min each time. AMP2 to AMP6 were added successively, and wash buffer twice before each addition, for 2 min each time. (6) Color display: Fast Red B and Fast Red A in RNAscope 2.5 HD Reagent Kit-Red assay Kit were added to sections with a ratio of 1:60. Reaction was performed at room temperature for 3–5 min, and observed under microscope until Red signal was detected. The slices were put into dH_2_O to terminate the reaction; (7) hematoxylin restaining: put the slices into the hematoxylin dye solution for about 5 s for restaining, and clean with tap water for 6–8 times, and finally change to dH_2_O for cleaning again. (8) Image acquisition and analysis.

### Western blotting was used to analyze the protein expression changes of Wnt10a, Axin2, β-catenin, and DSPP

Total cellular proteins were extracted using RIPA lysate and proteins were quantified by using the BCA protein assay kit. Samples were electrophoresed through sodium dodecyl sulfate–polyacrylamide gels and transferred to polyvinylidene difluoride membranes. Using β-actin as an internal reference control and sealing with 5% fat-free milk, the primary antibody was incubated: Axin2 antibody (1:1000), β-catenin antibody (1:1000), Wnt10a antibody (1:1000), DSPP antibody (1:2000), and mouse anti-β-actin antibody (1:5000). Then the membrane was incubated with the secondary antibody (goat anti-mouse IgG). Development and exposure were performed. Analyzed protein bands in grayscale were used with Image J software and relative protein expression was calculated.

### Isolation and identification of mouse dental pulp stem cells

The incisors of mice were collected and rinsed with PBS containing 5% diamantine for 2–3 times until there was no obvious blood clot on the tooth surface. Part of the tooth tissue of the tooth cusp and root tip was cut until the pulp point was visible and exposed. Then the pulp in the tooth was flushed out with MEM base medium through a 1-ml syringe, and the pulp in the pulp cavity was removed and then rinsed again. The rinse solution was collected and centrifuged at 1000 rpm/5 min, and the precipitate after centrifugation was pulp tissue (Appendix Fig. 8). Large pieces of pulp tissue were cut up and digested with a mixture of type I collagenase and Dispase II at 37℃. This was followed by culturing with MEM medium containing 20% FBS. The P3-generation DPSCs were identified by flow cytometry after staining by adding 5 µl of FITC fluorescently labeled CD44, CD45, CD105, CD34, CD146, and CD31 (Appendix Fig. 10(*A*, *B*)), under light-protected conditions, respectively (Almeida et al. 2011; Leong et al. 2012; Sakai et al. 2012; Yamamoto et al. 2014).

### Osteogenesis, lipogenesis, and chondrogenesis induction and identification of dental pulp stem cells

Osteogenesis induction and identification: P3 generation mice dental pulp stem cells were inoculated into coated Petri dishes at a cell density of 2 × 10^4^ cells/cm^2^, cultured at 37℃ with 5% CO_2_ to a confluence of 60–70%. Then we discarded liquid supernatant and added osteogenesis-induced differentiation medium (osteogenesis-induced differentiation basal medium 175 ml, specialized fetal bovine serum 20 ml, glutamine 2 ml, dual antibody 2 ml, ascorbic acid 400 µl β-glycerophosphate sodium 2 ml, dexamethasone 20 µl). About 14 days later, the termination time of cell induction was determined according to the precipitation of intracellular calcium salt crystals and the formation of calcium nodules, and identified by Alizarin Red Staining (Appendix Fig. 10(*C*)).

Lipogenesis induction and identification: P3-generation mouse dental pulp stem cells were inoculated, and lipogenesis-induced differentiation medium solution A (lipogenesis-induced differentiate-on basal medium A 175 ml, FBS 20 ml, glutamine 2 ml, dual antibodies 2 ml, insulin 400 µl, IBMX 200 µl, rosiglitazone 200 µl, dexamethasone 200 µl) and B solution (lipogenic induction differentiation basal medium B 175 ml, FBS 20 ml, dual antibodies 2 ml, glutamine 2 ml, insulin 400 g) were added to the basal mediums respectively, then the medium was mixed well and placed in the warmed bath. Lipogenic cells were induced by adding the configured lipogenic A solution, changing the B solution once after 3 days, keeping it for 1 day and then changing the A solution. Osteogenic induction was added into osteogenic induction mediums, and we placed cells with the added induction medium in the incubator and continued to culture for 14 d. Depending on the condition of the cells, the time to finish the cell induction was decided and Oil Red O Staining was performed for identification (Appendix Fig. 10(*D*)).

Chondrogenesis induction and identification: Using the instructions of the chondrogenesis induction kit, P3-generation mouse dental pulp stem cells were digested and counted at 3 × 105cells in 15-ml centrifuge tubes, respectively, and centrifuged at 220 g for 8 min at low speed to allow the cells to form microclusters. The cells were cultured in centrifuge tubes with chondrogenic induction solution, and we changed liquid at 3–4-d intervals, induced for 5 weeks, and fixed the cells in 4% paraformaldehyde, and paraffin sections were made routinely. The sections were dewaxed in xylene, dehydrated in gradient ethanol, and washed in distilled water before being stained with Alcinium Stain for identification (Appendix Fig. 10(*E*)).

### EDU staining was used to detect the changes in the proliferation of dental pulp stem cells after R-spondin, LiCl, and DKK1 treatment

The P3 generation dental pulp stem cells were digested. After centrifugation, we discarded supernatant liquid, washed them twice in PBS buffer, counted and transferred them to a 6-well plate with the corresponding volume of cell suspension, and then incubated them with 0.75 g/L LiCl, 1 µg/ml DKK1, and 1 µg/ml R-spondin for 24 h. According to BeyoClick EDU-488 cell proliferation detection reagent instructions, the cells were EDU labeled, fixed, washed, and permeated, then prepared the Click Additive Solution and 0.5 ml Click solution was added to each well, uniformly coated and incubated at room temperature for 30 min, protected from light. The Click reaction solution was removed by suction, and the cells were washed 3 times with PBS containing 3% BSA for 3–5 min each time. After nuclear staining with 1 × Hoechst 33,342 solution, the proliferation of cells in different groups could be analyzed by fluorescence detection.

### Wnt10a-siRNA transfection

Design Wnt10a-siRNA sequence sense primer was 5′UGGAUUUGUACCAUUCUUCUG 3′; anti-sense primer was 5 ′GAAGAAUGGUACAAAUCCAAG 3′ and synthesis of Wnt10a-siRNA. The day before transfection, P3 generation DPSCs were inoculated into 6-well plates at a density of 4 × 10^5^/ml with 2 ml/well and added 1.5 ml fresh serum-free DMEM medium. For each transfected well, a mixture of Wnt10a-siRNA and PEI40K Transfection Reagent was prepared as follows: 50 nM Wnt10a-siRNA was added into 100 µl serum-free DMEM medium and gently mixed. Then 6 µl of PEI40K Transfection Reagent was diluted in100µl growth medium and incubated at room temperature for 5 min. Dilute Wnt10a-siRNA and PEI40K Transfection Reagent were mixed with and incubated at room temperature for 20 min. Add the above mixture to a 6-well plate containing P3 generation DPSCs, with a final volume of 2 ml per well, and gently shake and mix. The cells were cultured in a carbon dioxide incubator at 37℃ for 6 h. The DMEM medium containing serum was changed and the cells were cultured for 48 h.

### Statistical analysis

In this study, SPSS 19.0 software was used to statistically analyze the data and the measurement data were expressed as (x ® ± s). Independent sample *t*-test was used for inter-group comparison, and paired *t*-test was used for intra-group comparison. Enumeration data were expressed as a rate (%) and compared by *X*^2^ test. *P* < 0.05 was considered statistically significant.

## Results

### LiCl intervention can promote the repair of different types of dentin-pulp injury in adult mice incisors, while DKK1 inhibited this process; the cutting method requires slightly longer repair time than the abrasion method

The root of the incisor on one side was abraded by the abrasion method until the red pulp piercing point was visible to the naked eye. The diameter of defect in the abrasion model group, abrasion LiCl group, and abrasion DKK1 group was 1.0–1.5 mm, and the depth was about 1.0 mm (Fig. 1(A1, A1')). In the cutting method, one side of the mouse incisor tooth was directly cut with sterilized ophthalmic scissors until the broken end of the incisor tooth was horizontal with the lingual gingiva, and the red pulp penetration point was visible to the naked eye (Fig. 1(A2, A2')). The injured and medicated incisor teeth were observed continuously for 7 d, and the repair of the incisor teeth was recorded (Fig. 1(*B*, *C*)). The repair of the tooth surface and interior was observed by gross morphology and micro-CT, and taking the control group and the contralateral incisor of the injured group as reference, the length of the teeth repaired was consistent with that of the opposite side, and the surface of the teeth was smooth without defects, and micro-CT showed that the basic structure of the internal teeth was consistent with the control group and the contralateral teeth of the injured group, which was judged as the completion of repair. With 7 days as the observation period, the recovery rate of the abrasion LiCl group was 100% in 7 d, and the average recovery time was 5.2 d. The recovery rate of the cutting LiCl group was 100% after 7 d, and the average recovery time was 6.5 days. The recovery rate of the abrasion DKK1 group was 30% after 7 d, and that of Cutting DKK1 group was 20%. The results showed that LiCl intervention promoted the repair of dentin-pulp injury, while DKK1 inhibited the repair process, and the repair time in the abrasion group was shorter than that in the cutting group.

### The injury can promote the proliferation of DPSCs and the differentiation of odontoblast cells, which can be accelerated by LiCl intervention while inhibited by DKK1

Incisor sections prepared by the EDTA decalcification method were stained with Ki67 immunofluorescence, observing the changes in cell proliferation in the incisor dentin-pulp injury model treated with LiCl and DKK1 for 7 d.

**Figure. 2:**
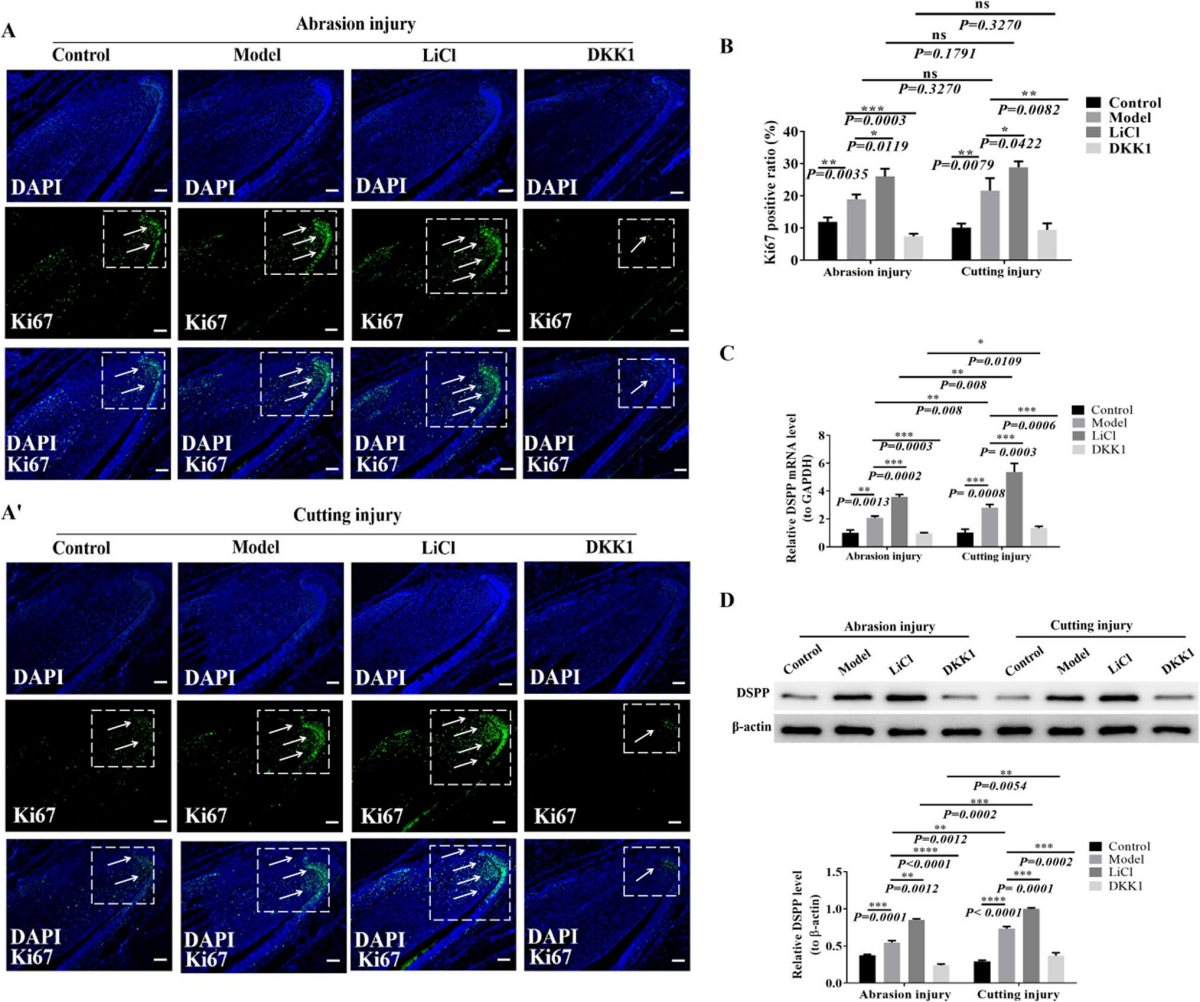
The changes in proliferation and odontoblast differentiation of DPSCs were detected and analyzed in different injury models of mouse incisors. (*A*, *A*') The expression of Ki67 in incisor tissue was detected by immunofluorescence staining in the abrasion and cutting groups. The *white arrows* show Ki67 positive cells. The Ki67-positive cells were expressed in the cervical ring of the incisor teeth of mice in the control group. The expression of Ki67 in the model group was significantly higher than in the control group. And compared with the control group, Ki67 expression increased more significantly in the LiCl group and decreased prominently in the DKK1 group. *Scale bars*, 100 μm. (*B*) The ratio of Ki67 positive cells in the abrasion, cutting, control, model, LiCl, and DKK1 groups were analyzed respectively. (*C*) After 7 days of LiCl and DKK1 induction, the qRT-PCR analysis of DSPP mRNA (to GAPDH) in the abrasion model, abrasion LiCl, abrasion DKK1, cutting model, cutting LiCl, and cutting DKK1 groups. *n* = 70, *P < 0.05, **P < 0.01, ***P < 0.001. (*D*) Western blotting analysis of DSPP expression in the abrasion model, abrasion LiCl, abrasion DKK1, cutting model, cutting LiCl, and cutting DKK1 groups after 7 d of LiCl and DKK1 induction. *n* = 70, *P < 0.05, **P < 0.01, ***P < 0.001.

The number of Ki67 immunofluorescence–stained positive cells in the abrasion model and cutting model groups were significantly higher than those in the control group. It was suggested that incisor injury could stimulate the proliferation of DPSCs, but there was no statistical significance between the two injury models (Fig. 2(*B*)). However, compared with the control group and the model group, Ki67 positive cells in the drug group showed different results. After LiCl injection, the number of Ki67 positive cells increased significantly. The number of Ki67-positive cells decreased markedly after DKK1 injection (Fig. 2(*A*, *A*’)).

The qRT-PCR was used to detect the expression of salivary phosphoprotein (DSPP) mRNA in damaged dentine models after 7 days of LiCl and DKK1 intervention; it was found that DSPP mRNA levels increased after abrasion and cutting injury compared with the control group (Fig. 2(*C*)). The expression of DSPP mRNA in the cutting model group and cutting LiCl group was higher than that in the abrasion model group and abrasion LiCl group, and the difference was statistically significant. These results suggest that DPSCs can be stimulated to differentiate into odontoblast cells after incisor dentin-pulp injury, and there is a significant relationship between the degree of incisor injury. Compared with the two injury model groups, the expression level of DSPP mRNA in LiCl injection group was further increased. On the contrary, the mRNA expression level of DSPP decreased significantly in the DKK1 injection group (Fig. 2(*D*)). The studies (Heo et al. 2010; Wang et al. 2014; Neves et al. 2017; Chatvadee et al. 2022) have shown that LiCl and DKK1 are activators and inhibitors of Wnt/β-catenin signaling pathway, combined with the above results; it is speculated that the changes of DPSC proliferation and odontoblasts differentiation after injury may be closely related to the changes of Wnt/β-catenin signaling pathway.

### Wnt10a and Wnt/β-catenin signaling pathways play an essential role in different types of repair of dentin-pulp injury

**Figure. 3:**
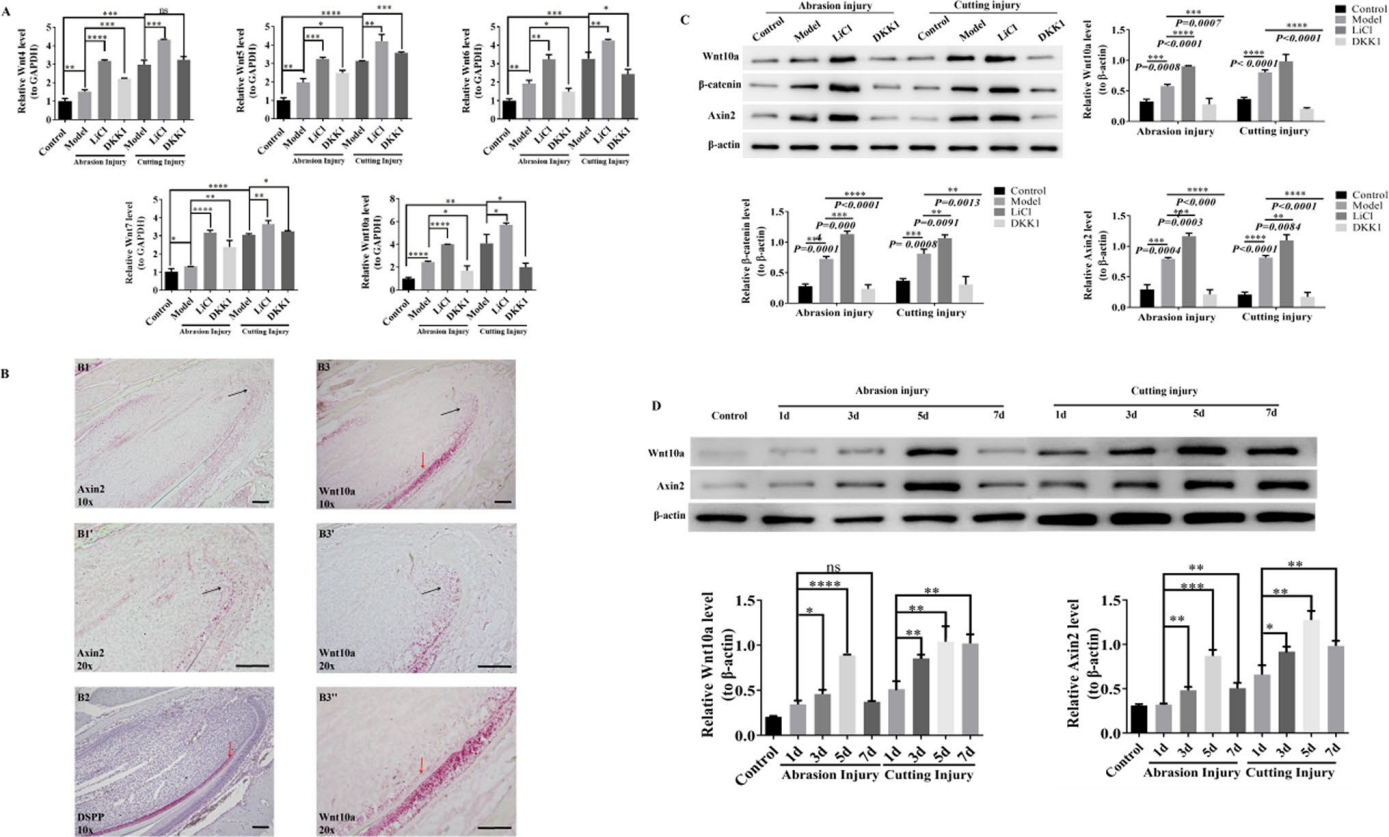
Detection of Wnt4, Wnt5, Wnt6, and Wnt7 and changes in the expression levels of Wnt10a and Wnt/β-catenin signaling pathways in mouse incisors of different injury models, as well as changes in Wnt10a and Wnt/β-catenin signaling pathways at different time points after injury. (*A*) The expressions of Wnt4, Wnt5, Wnt6, Wnt7, and Wnt10a in the incisor were detected by qRT-PCR. *P < 0.05, **P < 0.01, ***P < 0.001. *Scale bars*, 100 μm. (*B*) RNAscope in situ hybridization for the expression of Wnt10a and Axin2 in normal mouse incisors. The *scale bars* in the figure are all 100um. (B1) Under a 10 × microscope, Axin2 appeared high expression in the proliferative zone, and the *black arrow* and pink staining are positive for Axin2. (B1') was a magnification of (B1) at 20 × microscope in the *black arrow* region. (B2) DSPP appeared high expression in the odontoblast region, and the *red arrow* and pink staining are DSPP positive. (B3) Under a 10 × microscope, Wnt10a appeared high expression in the proliferative zone, with rarely expressed in the odontoblast zone. *Black arrow*, *red arrow*, and pink staining represent the presence of Wnt10a positivity in the proliferative and odontoblast regions, respectively. (B3') and (B3'') are (B3) at 20 × electron microscope with magnification of the black and red arrow areas, respectively. (*C*) Western blotting detected the expression of Wnt10a, β-catenin, and Axin2 in the control group, abrasion model group, abrasion LiCl group, abrasion DKK1 group, cutting model group, cutting LiCl group, and cutting DKK1 groups. (*D*) Western blotting detected changes in the expression of Wnt10a and Axin2 at 1, 3, 5, and 7 d after incisor abrasion and cutting injuries.

Previous researches have been shown that Wnt4, Wnt5, Wnt6, Wnt7, and Wnt10a are observed to express in the dental epithelium and mesenchyme during early tooth development (Lin et al. 2011; Wang et al. 2014; Miyazaki et al. 2019). In order to further study the Wnt family members and clarify the role of Wnt/β-catenin signaling pathway in the repair of dentin-pulpal injury, we combined the literature, and used qRT-PCR to detect the expression of Wnt4, Wnt5, Wnt6, Wnt7. and Wnt10a in the incisor tissues of our seven groups. The results revealed that the downregulation changes of Wnt4, Wnt5. and Wnt7 were not obvious with the intervention of DKK1, although Wnt6 has a certain reduction, but not as obvious as the change of Wnt10a (Fig. 3(*A*)). Meanwhile, our preliminary experiment (Shi et al. 2019) used RNAScope in situ hybridization to observe the incisal sections of the control group and found that Wnt10a was mainly expressed in the cervical ring region, which coincided with the regions expressed by Ki67 and Axin2, and partially expressed in the odontoblast region, which coincided with the region expressed by DSPP (Fig. 3(*B*)). Therefore, we selected Wnt10a for further study.

We used Western blotting and qRT-PCR to detect the expression of Wnt10a, β-catenin, and Axin2 in incisor tissues of seven groups to observe the expression of Wnt10a in the injured pulp tissue and the effect of LiCl and DKK1 on it. Compared with the control group, the protein and mRNA expression of Wnt10a, β-catenin, and Axin2 were significantly higher in both model groups. Compared with the model group, the protein and expression of Wnt10a, β-catenin, and Axin2 were further elevated in the LiCl group. In contrast, Wnt10a, β-catenin, and Axin2 protein expression were significantly decreased in the DKK1 group (Fig. 3(*C*)). These results suggest that Wnt10a and Wnt/β-catenin signaling pathways can be activated by different types of dentin-pulp complex injuries, and stimulation with LiCl increased Wnt10a expression and upregulated Wnt/β-catenin signaling pathway, while the intervention with DKK1 produced the reverse results.

In order to clarify the changes of Wnt10a and Wnt/β-catenin signaling pathway at different times after injury, the expression of Wnt10a and Axin2 at 1, 3, 5, and 7 d after injury was detected by Western blotting. The results showed that the expression of Wnt10a and Axin2 increased with the increase of days from 1 to 5 days after injury in both the abrasion group and the cutting group. And the expression of Wnt10a and Axin2 was the most obvious on day 5 after injury (Fig. 3(*D*)). However, on 7 days after injury, the expression of Wnt10a and Axin2 was still pronounced in the cutting group, but the expression was significantly reduced in the abrasion group and approached to the control group. It was suggested that Wnt10a and Wnt/β-catenin signaling pathway were closely related to dentin-pulp injury repair. It is also speculated that Wnt10a may participate in the repair process by mediating Wnt/β-catenin signaling pathway.

### Cell experiments confirmed that LiCl promotes the proliferation and dentinogenic differentiation of DPSCs through upregulation of Wnt10a and Wnt/β-catenin signaling pathways, whereas DKK1 produced opposite results

Flow cytometry assays and induction of lipogenesis, osteogenesis, and chondrogenesis toward differentiation of cultured cells showed that the cultured cells were positive for the dental pulp stem cell mesenchymal markers CD44, CD105, and CD146, and negative for the hematopoietic markers CD45, CD34, and CD31, and there was significant induction and differentiation of lipogenesis, osteogenesis, and chondrogenesis. It is shown that the isolated cultured mouse DPSCs had good properties of stem cells (Appendix Fig. 10(*A*)).

**Figure. 4:**
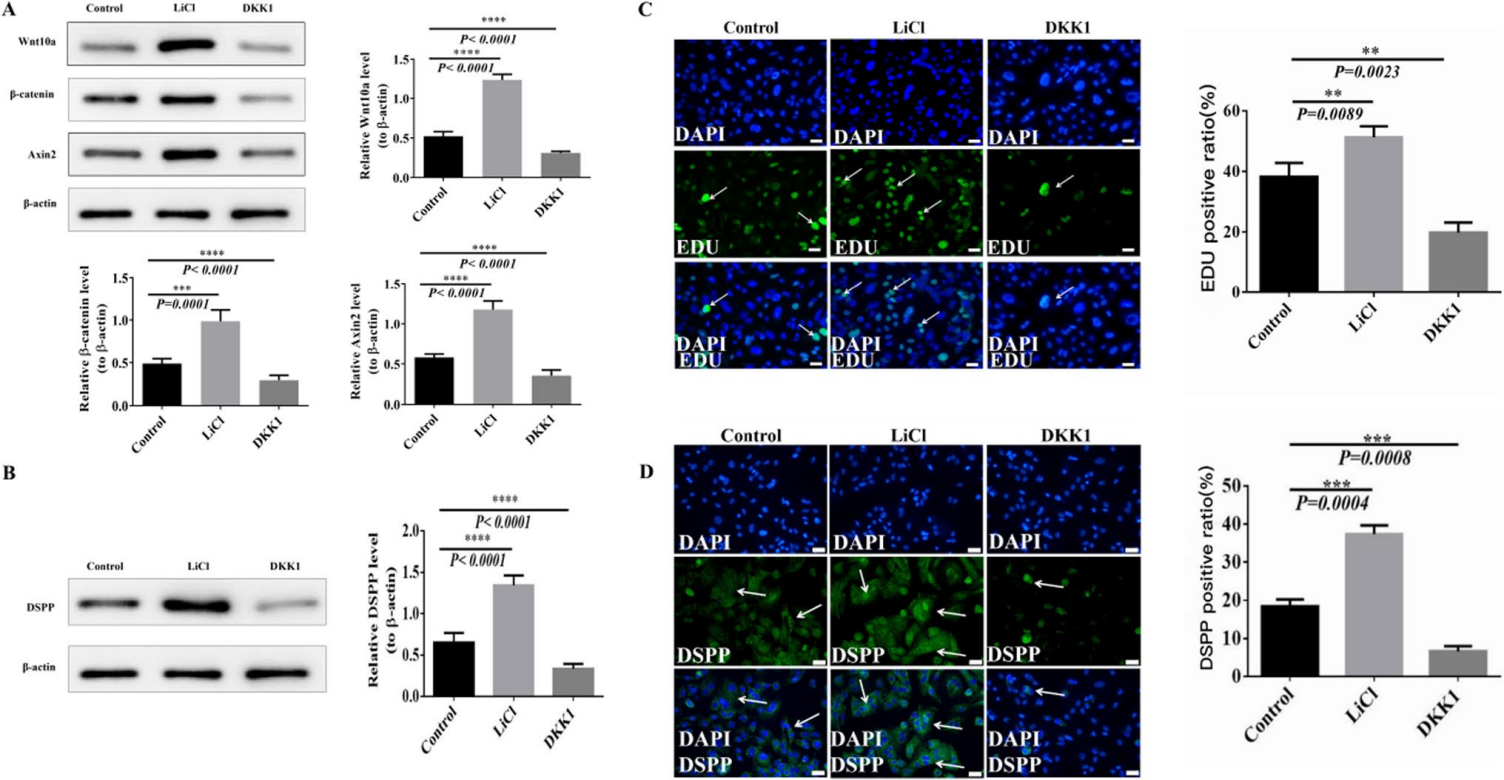
In the cell experiment, Western blotting detected the changes in the expression levels of Wnt10a, Wnt/β-catenin signaling pathway, and DSPP stimulated by LiCl and DKK1, and EDU and DSPP staining were used to analyze the proliferation and odontoblast differentiation of DPSCs and the differentiation of odontoblast cells. *P < 0.05, **P < 0.01, ***P < 0.001. *Scale bars*, 50um. (*A*) Western blotting analyzed expression changes of Wnt10a, β-catenin, and Axin2 proteins in the control, LiCl, and DKK1 groups under the stimulation of LiCl and DKK1. (*B*) Western blotting analyzed expression changes of DSPP protein in control, LiCl, and DKK1 groups under the stimulation of LiCl and DKK1. (*C*) EDU staining was used to observe the proliferative vitality of DPSCs in P3 generation mice stimulated by LiCl and DKK1. *White arrows* and green staining are positive cells. The proportion of positive cells in the control, LiCl, and DKK1 groups were analyzed. (*D*) DSPP immunofluorescence staining was used to identify mice DPSCs stimulated by LiCl and DKK1. *White arrows* and green staining are positive staining of DSPP. The positive proportion of DSPP in the control, LiCl, and DKK1 groups were analyzed.

Compared with the control group by Western blotting, the expression of Wnt10a, β-catenin, Axin2, and DSPP proteins were found to be significantly higher in the LiCl group and markedly lower in the DKK1 group (Fig. 4*A*, *B*). Then, the proliferation and odontoblast differentiation of DPSCs that interfered with LiCl and DKK1 were observed by immunofluorescence staining with EDU and DSPP, respectively. EDU and DSPP staining positive cells could be observed in the control, LiCl, and DKK1 groups. However, there were significant differences between three groups, with more EDU and DSPP staining positive cells in the LiCl group than in the control group, while the DKK1 group was significantly lower than the control group (Fig. 4*C*, *D*). These results verified that LiCl promoted the proliferation and odontoblast differentiation of DPSCs by upregulating Wnt10a and Wnt/β-catenin signaling pathways, but DKK1 produced opposite results.

### Wnt10a plays an essential role in dentin-pulp repair by mediating the Wnt/β-catenin signaling pathway

**Figure. 5:**
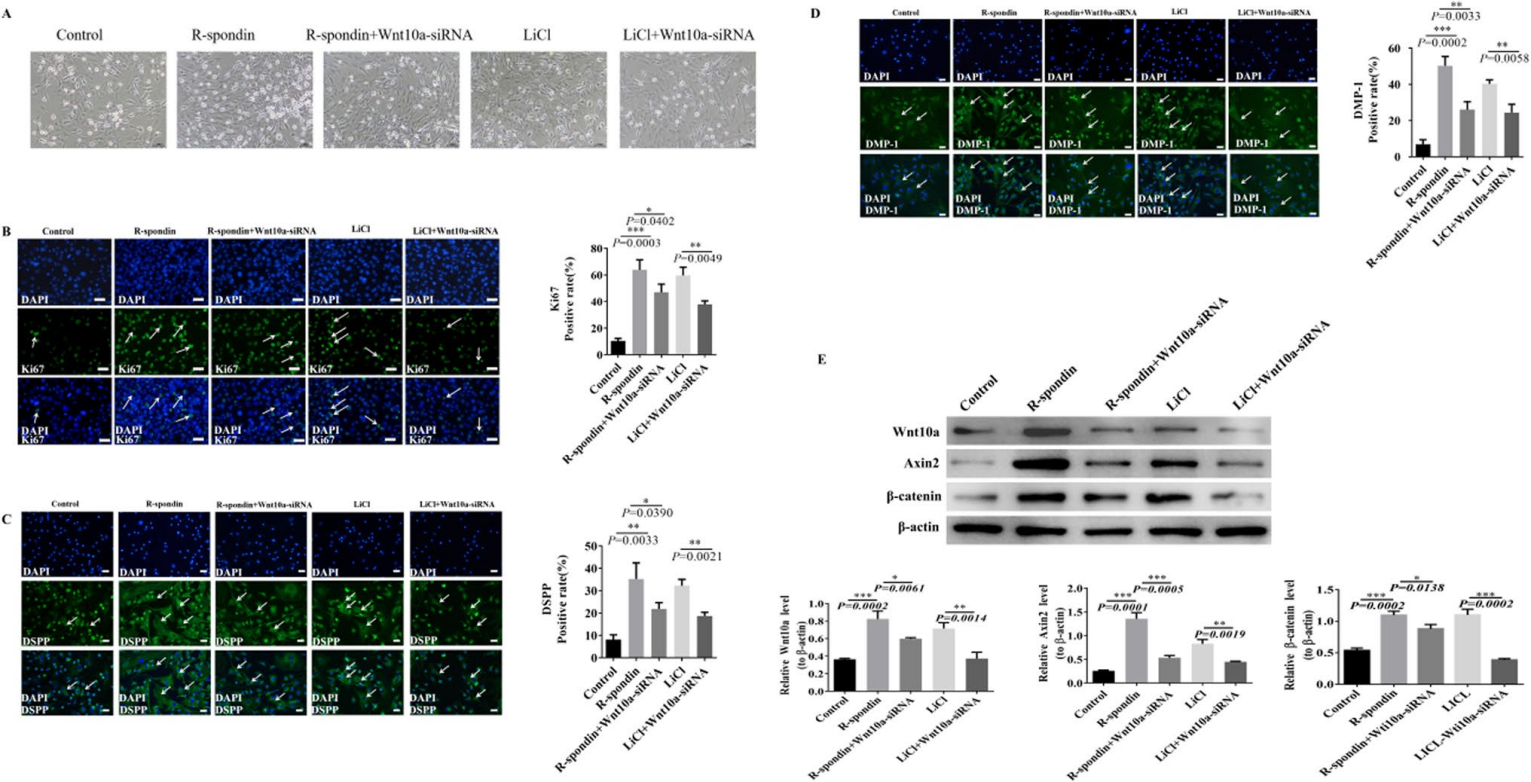
Normal DPSCs and Wnt10a-siRNA DPSCs of mice were interfered by R-spondin and LiCl. Ki67 fluorescence staining, DSPP, and DMP-1 immunofluorescence staining were used to detect the changes of DPSC proliferation and odontogenesis differentiation of normal DPSCs and Wnt10A-SiRNA, and Western blotting was used to detect the changes of Wnt10a, Axin2, and β-catenin protein expression. *P < 0.05, **P < 0.01, ***P < 0.001. *Scale bars*, 50 µm. (*A*) Cell growth was observed in normal mice DPSCs and Wnt10a-siRNA DPSCs after R-spondin and LiCl intervention by microscope. (*B*) Ki67 fluorescence staining detected the proliferative vitality of DPSCs in control, R-spondin, LiCl, R-spondin + Wnt10a-siRNA, and LiCl + Wnt10a-siRNA groups. *White arrows* and green staining are positive cells, and analyzing the proportion of positive cells in each group. (*C*) Fluorescence staining detected DSPP in control, R-spondin, LiCl, R-spondin + Wnt10a-siRNA and LiCl + Wnt10a-siRNA groups. *White arrows* and green staining are DSPP positive cells, and analyzing the proportion of positive cells in each group. (*D*) Fluorescence staining detected DMP-1 in control, R-spondin, LiCl, R-spondin + Wnt10a-siRNA group, and LiCl + Wnt10a-siRNA group. *White arrows* and green staining are DMP-1 positive cells, and analyzing the proportion of positive cells in each group. (*E*) Western blotting analysis of Wnt10a, Axin2, and β-catenin protein expression in Control, R-spondin, LiCl, R-spondin + Wnt10a-siRNA, and LiCl + Wnt10a-siRNA group.

In order to confirm that Wnt10a is involved in the repair of dentin-pulp injury by mediating the Wnt/β-catenin signaling pathway, we constructed Wnt10a-siRNA DPSCs for the study. Previous research has shown that R-spondin is an activator of Wnt/β-catenin signaling pathway (Chatvadee et al. 2022). We used R-spondin and LiCl respectively to activate the Wnt signaling pathway in normal DPSCs and Wnt10a-siRNA DPSCs to double confirm the activation of Wnt/β-catenin signaling pathway in the cell experiment, and we divided them into the control, R-spondin, R-spondin + Wnt10a-siRNA, LiCl, and LiCl + Wnt10a-siRNA groups. In the observation of cell culture under the microscope, the result showed that compared with the control group, the number of DPSCs in R-spondin and LiCl groups increased, and compared with the R-spondin and LiCl groups, the number of DPSCs in R-spondin + Wnt10a-siRNA group and LiCl + Wnt10a-siRNA group decreased significantly (Fig. 5*A*). Ki67 staining was used to observe the proliferation of DPSCs after R-spondin and LiCl intervention, and immunofluorescence staining was used to detect DSPP and DMP-1 to analyze odontoblast differentiation of DPSCs after R-spondin and LiCl stimulation (Fig. 5*B*, *C*, *D*). The results showed that compared with the control group, Ki67 positive cells, DSPP, and DMP-1 positive cells were significantly increased after using R-spondin and LiCl, indicating that the Wnt/β-catenin signaling pathway was activated by R-spondin and LiCl, and the proliferation and odontoblast differentiation of DPSCs were also significantly enhanced. Compared with the R-spondin group and the LiCl group, Ki67 positive cells, DSPP, and DMP-1 immunofluorescence positive cells in the R-spondin + Wnt10a-siRNA group and the LiCl + Wnt10a-siRNA group were significantly reduced; these suggested that activation of the Wnt/β-catenin signaling pathway stimulates a significant increase in the proliferation and odontoblast differentiation of DPSCs, while interference with Wnt10a diminished the proliferation and odontoblast differentiation of DPSCs, and Wnt10a plays an important role in the restorative process by mediating the Wnt/β-catenin signaling pathway.

To further confirm the speculation, we used Western blotting to detect the expression of Wnt10a, Axin2, and β-catenin proteins in the R-spondin, R-spondin + Wnt10a-siRNA, LiCl, and LiCl + Wnt10a-siRNA group and the results showed that compared with the control group, the expression of Wnt10a, Axin2, and β-catenin was elevated both in the R-spondin and LiCl groups (Fig. 5*E*), indicating that both R-spondin and LiCl could activate Wnt10a and Wnt/β-catenin signaling pathway. After interfering with Wnt10a, the expression of Wnt10a, Axin2, and β-catenin was significantly decreased in the R-spondin + Wnt10a-siRNA group and the LiCl + Wnt10a-siRNA group. This indicated that the Wnt/β-catenin signaling pathway was inhibited after interference with Wnt10a, confirming that Wnt10a plays an essential role in the repair process of pulp-dentin complex damage by mediating the Wnt/β-catenin signaling pathway.

## Discussion

The restoration and regeneration of injured teeth has always been a hot topic in dental research, and the construction of injured teeth model is the primary problem to be solved. When a tooth is injured by dental caries, mechanical abrasion, or trauma, the inflammatory response can stimulate pulp stem cells to perform their repair function. In this study, abrasion and cutting methods were used to study, aiming to simulate dental caries, iatrogenic pulp perforation, and trauma resulting in different areas of dentin-pulp complex injury in clinical practice severally, so as to more comprehensively analyze the repair situation after injury under different circumstances (Wang et al. 2014; Zhong et al. 2019). After dental defects caused by caries or trauma, it is difficult for human incisors and molars to repair themselves. However, mesenchymal stem cells are still retained in the incisor teeth of mice to the adult stage, which can effectively promote injured repair (Sønstevold et al. 2015), which providing a good model for our research. The repair and regeneration of mice incisor after injury including enamel, dentin and pulp, and the regeneration of enamel mainly comes from epithelial stem cells, and the regeneration of dentin and pulp mainly comes from mesenchymal stem cells. Because of the dental pulp includes vascular bundles, nerve bundles, and a variety of cell groups, it is complicated to study the repair and regeneration of dental pulp alone. Therefore, this study focused on the differentiation of DPSCs into odontoblast cells and the repair and regeneration of dentin-pulp complex, laying a certain research foundation for the cultivation of “organoids” in the future.

The repair of dentin-pulp injury is closely related to the regulation of signal pathway. The Wnt/β-catenin signaling pathway has been shown to play an important role in tooth development, including cell fate differentiation, polarization, migration, and cell proliferation. Wnt4, Wnt5, Wnt6, Wnt7, and Wnt10a expressed in dental epithelium and mesenchyme at the early stage of tooth development (Wang et al. 2014). Zhong et al. (2019) found that the inflammatory condition of dental pulp would reduce the expression of Wnt4 in DPSCs, thus inhibiting the odontoblast differentiation of DPSCs. Lin *et al*. (2011) found that the absence of Wnt5a and Ror2 led to delayed development of mice dental crowns, impaired differentiation of odontoblast cells, and dental crown malformations. Cheng et al. (2010) found that Wnt6 played an important role in promoting the differentiation of hDPSCs, but had no significant effect on their proliferation. The study of Chen et al. (2019) found that Wnt7b was observed in the odontoblast and ameloblast layers, indicating that it may play a role in the differentiation of odontoblast and ameloblast. Previous studies on Wnt10a were mainly related to the abnormal formation of root bifurcation, the individual loss of teeth, and congenital malformation (Park et al. 2019; Yu et al. 2020). In Fig. 3(*A*) of this study, qRT-PCR results showed that the expression of Wnt4, Wnt5, and Wnt7 in the DKK1 group was all higher than that in the model group, but DKK1 was an inhibitor of Wnt signaling pathway, and the result was contrary to the research on the related mechanism of Wnt signaling pathway. When the Wnt/β-catenin signaling pathway was activated in the abrasion and cutting groups, the expressions of Wnt6 and Wnt10a were increased due to injury and LiCl stimulation, while that were decreased due to the inhibition of DKK1, but the change of Wnt10a was more obvious than that of Wnt6, suggesting that Wnt10a may play a more important role. In addition, our previous study found that the expression regions of Wnt10a, Axin2, and Ki67 in normal mice incisor teeth were consistent, suggesting that their effects were closely related to proliferation; Wnt10a was also expressed in the odontoblast region, suggesting that Wnt10a was also related to the differentiation of odontoblast cells. However, its role in the repair of dentin-pulp injury is unclear, and there are fewer comparative studies among different injury models. Therefore, we choose Wnt10a as the research gene to further explore the mechanism of Wnt10a in the repair of different dentin-pulp injury.

In this study, the dentin-pulp injury model of mice incisor was successfully constructed by abrasion and cutting method respectively. The overall observation shows that the recovery time of cutting method is slightly longer than that of abrasion method, which may be related to the injured area, and the larger the injured area, the longer the repair time. Although the Wnt/β-catenin signaling pathway was activated by both abrasion and cutting methods to promote the proliferation of DPSCs and the differentiation of odontoblast cells, no thickening of dentin layer or narrowing of pulp cavity was observed in micro-CT. Considerations relate to the following aspects: Firstly, the increased odontoblast was mainly used for the repair of mice incisor teeth after injury. Secondly, because the injury is completed in a relatively short time, it will not cause the thickening of dentin layer. Thirdly, it may be related to the precise regulation of Wnt10a. According to Fig. 3(*D*), we found that the expression of Wnt10a continued to increase 1–5 d after injury, but on the 7th day after injury, the repair of the Abrasion group was basically completed; meanwhile, the expression of Wnt10a decreased, *basically* consistent with the control group. However, in the cutting group, Wnt10a was still significantly expressed on the 7th day after injury; we speculated that with the completion of injury repair, the expression of Wnt10a would also return to normal, rather than continuously high expression leading to the accumulation of odontoblast cells and the thickening of dentin layer. According to Fig. 2, on the 7th day after injury, there was no significant difference between different injury models in promoting the proliferation of DPSCs, but there was a significant difference between groups in promoting the differentiation of DPSCs odontoblast cells; the expression of DSPP in the abrasion group was slightly lower than that in the cutting group, and the expression of Wnt10a in the abrasion group was also lower than that in the cutting group, showing the same trend. In combination with Fig. 5*C*, it was found that the expression of DSPP decreased significantly after the addition of Wnt10A-siRNA, suggesting that Wnt10a may be the upstream gene of DSPP, which is consistent with the study of Zheng scholars (Zheng et al. 2007). Zhang et al. (2014) found that by transfecting human dearal pulpstem cells (hDPSCs) with lentivirus, the overexpression of Wnt10a could promote the proliferation of hDPSCs, but inhibit the differentiation of their odontoblast cells, which was inconsistent with the results of this study, considering that is related to the different disquisitive cells and experimental method, which also suggests the necessity of further research on the precise regulatory mechanism of Wnt10a in the future.

In recent years, some scholars believe that Wnt10a acts as a regulatory factor acting on the classical Wnt pathway (Park et al. 2019), but other scholars believe that Wnt10a acts through non-classical PCP and Wnt/Ca^2+^ pathway (Cao et al. 2021). In the dentin-pulp injury model, how Wnt10a plays a role in promoting repair and the specific regulatory mechanism is still unclear. In Fig. 3(*C*) and Fig. 4*A* of this study, it can be observed that LiCl can promote the expression of Wnt10a and activate the Wnt/β-catenin signaling pathway, while DKK1 can produce the opposite result. By constructing Wnt10a-siRNA DPSCs, in Fig. 5*E*, we found that compared with LiCl group, the protein expression levels of Wnt10a, Axin2, and β-catenin were significantly reduced in the LiCl + Wnt10a-siRNA group, suggesting that Wnt10a plays a role through mediating Wnt/β-catenin signaling pathway. Previous studies have found that LiCl and R-spondin are common activators of Wnt signaling pathway, but their action sites are different, LiCl inhibits GSK-3 to accumulate β-catenin, and then the β-catenin generates nuclear ectopia, then activating the transcription and expression of Wnt target genes (Neves et al. 2017). In contrast, R-spondin increases intracellular β-catenin by binding to Wnt receptor complex and enhancing Wnt signaling in the classical Wnt pathway (Kim et al.^2006^). In order to demonstrate that the Wnt/β-catenin signaling pathway was further activated by the increased expression of Wnt10a, we added R-spondin and LiCl for comparative study in subsequent studies and found that both of them could activate Wnt10a and Wnt/β-catenin signaling pathways. And after interfering with Wnt10a, the expressions of Wnt10a, Axin2, and β-catenin were significantly decreased in the LiCl + Wnt10a-siRNA group and the R-spondin + Wnt10a-siRNA group, confirming again that Wnt10a exerts an effect through the Wnt/β-catenin signaling pathway in this study. LiCl is generally thought to activate the Wnt/β-catenin signaling pathway by inhibiting GSK-3 and causing β-catenin accumulation. Interestingly, it was observed in this study that LiCl could promote the expression of Wnt10a, Axin2, and β-catenin and then initiate the Wnt/β-catenin signaling pathway. This suggests that LiCl has an activation effect on Wnt proteins, but the specific mechanism needs to be further explored.

In the process of dentin formation and mineralization, DSPP is a specific marker protein of odontoblast cells (Fitzgerald et al. 1990), and DMP-1 is an important matrix protein for dentin formation and mineralization (George et al. 1993). In the tissue sections and cell experiments, we detected the expressions of Ki67, DSPP, and DMP-1 with immunofluorescence staining, and analyzed the changes in proliferation and differentiation of odontoblast cells (Fig. 5); it was found that activation of Wnt10a and upregulated Wnt/β-catenin signaling pathway promoted the proliferation of DPSCs and differentiation of odontoblast cells. However, some scholars found that CCN3 also plays a role in the repair of dentin-pulp injury. Overexpression of CCN3 upregulates the Notch signaling pathway, which enhances the proliferation ability of DPSCs, but inhibits their odontogenic differentiation ability (Xuefei et al. 2014), suggesting that different factors and signaling pathways have different mechanisms of action.

## Conclusion

As a repair related protein, Wnt10a and Wnt10a-mediated Wnt/β-catenin signaling pathway plays a significant role in the repair of dentin-pulp injury, activating the expression of Wnt10a /β-catenin signaling pathway can promote the repair, and inhibiting the expression of this signaling pathway can prolong the repair time in both abrasion and cutting groups. The positive regulatory effect is realized by promoting the proliferation of DPSCs and the differentiation of odontoblast cells. Some studies have suggested that DPSCs are in the quiescent phase of the cell cycle after the completion of tooth development (Swaffer et al. 2016). When dentin and pulp are severely injured, DPSCs re-enter the cell cycle and stimulate the proliferation and differentiation of dentin cells to generate restorative dentin. However, whether the regulation of the signaling pathway on the proliferation of DPSCs and odontoblast differentiation after injury is related to the cell cycle still needs to be further explored. This study enriched the mechanisms of Wnt10a and Wnt/β-catenin signaling pathways in the repair of different types of dentin-pulp injury, which could provide experimental evidence for target genes screening and also give some new ideas for subsequent research on the molecular mechanisms related to tooth regeneration.

## Figures and Tables

**Figure. 6:**
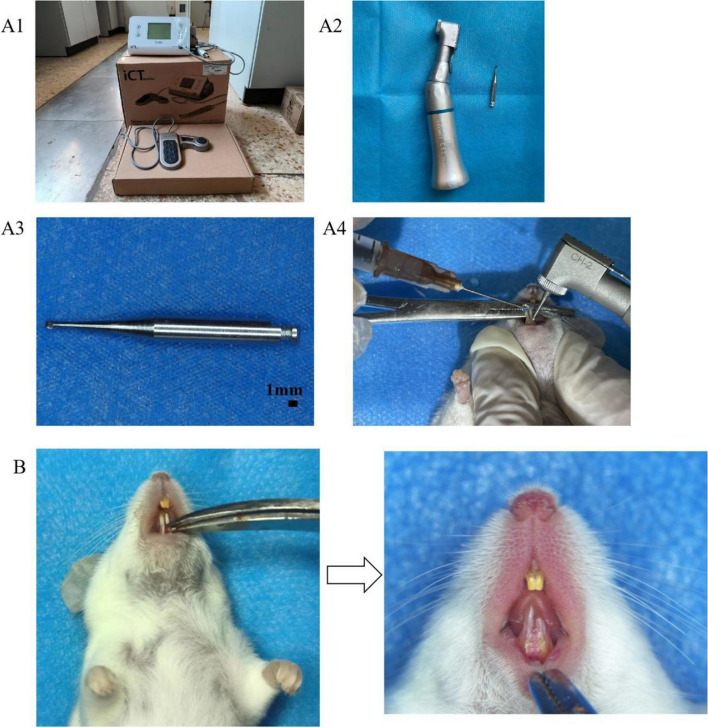
Model construction of mice incisor dentin-dental pulp damage. (*A*) The abrasion method: We used a dental slow-speed handpiece (EX-203C Set, NSK) connected to the planting machine power system (ICT motor Serial NO.B02DED1021, Dentium) and coupled with a 1-mm diameter tungsten steel slow bending ball drill (RA2, Dentsply Sirona) to abrade the root of one side incisor. During the abrasion process, the ball drill was stopped when the drill bit could fully enter the tooth tissue and the red pulp piercing point was visible to the naked eye. Cooling and washing with saline while grinding. The diameter of the defect was 1.0–1.5 mm and the depth was 1.0 mm. (A1) The planting machine power system (ICT motor Serial NO.B02DED1021, Dentium). (A2) Dental slow-speed handpiece (EX-203C Set, NSK). (A3) A 1-mm diameter tungsten steel slow bending ball drill (RA2, Dentsply Sirona). (A4) The process of abrasion. (*B*) The cutting method: One side incisor was cut directly with ophthalmic scissors until the broken end of the incisor was horizontal with the lingual gingiva.

**Figure. 7:**
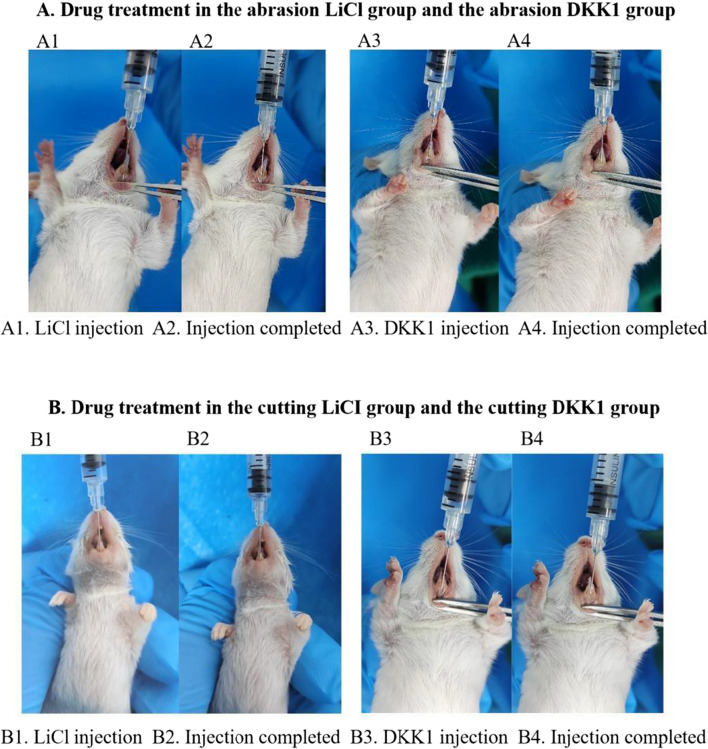
Drug treatment in the abrasion groups and the cutting groups. For the periodontal ligament injection method, a needle was inserted into the gingival sulcus on the labial side of the injured incisor and normal incisors on one side of the control group, the inclined surface of the needle was close to the tooth surface, and the needle direction was at an angle of 45° to the long axis of the tooth, then slowly penetrated the periodontal membrane, and the drug was injected. The mice in the abrasion LiCl group and the cutting LiCI group were given LiCl solution (0.75 g/L) at a dose of 0.2 ml/kg and The mice in the abrasion DKK1 group and the cutting DKK1 group were injected with DKK1 protein (1ug/ml) at a dose of 0.2 μg/kg by local injection into the periodontal ligament, once every 2 d. (*A*) Drug treatment in the in the Abrasion LiCI group and the abrasion DKK1 group. (A1–A4) Process of drug treatment. (*B*) Drug treatment in the in the cutting LiCI group and the cutting DKK1 group. (B1–B4) Process of drug treatment.

**Figure. 8:**
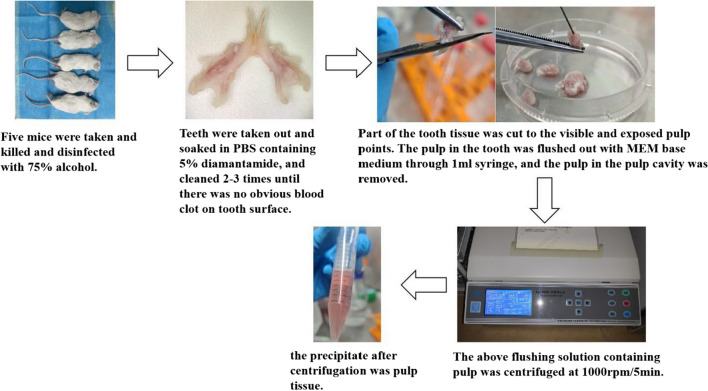
Sampling method of mouse dental pulp. Five mice were sacrificed by excessive carbon dioxide and cervical dislocation. Then we put them in 75% alcohol to disinfect for 5 min. The mandibles were isolated and the soft tissues attached to the mandibles were removed and the mandibles were rinsed with PBS containing 5% diamantine for 2–3 times until there was no obvious blood clot on the tooth surface. Part of the tooth tissue of the tooth cusp and root tip was cut until the pulp point was visible and exposed. Then the pulp in the tooth was flushed out with MEM base medium through a 1-ml syringe and the pulp in the pulp cavity was removed, then rinsing again. The rinse solution was collected and centrifuged at 1000 rpm/5 min, and the precipitate after centrifugation was pulp tissue.

**Figure. 9:**
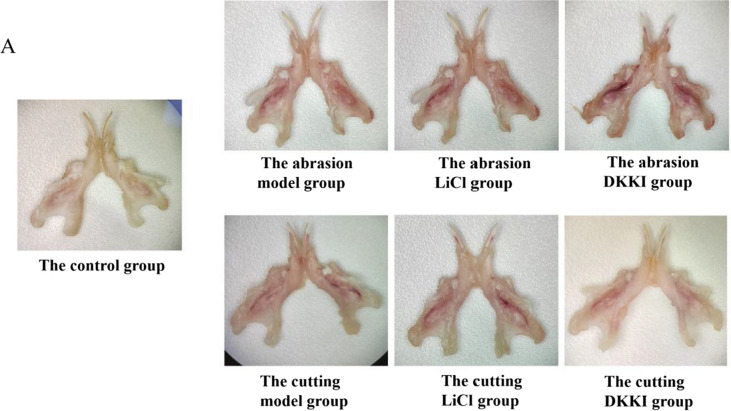

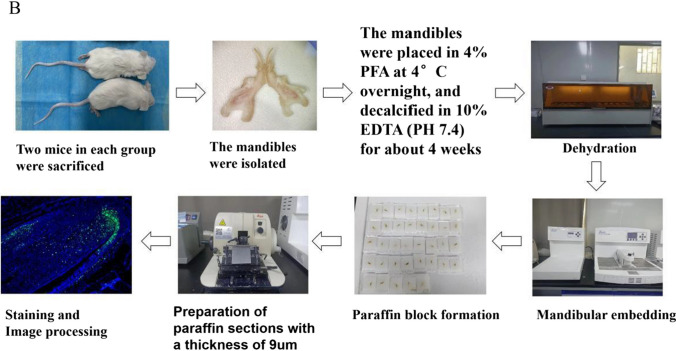
Preparation of paraffin sections. Two mice in each group were sacrificed, the mandibles were isolated and the soft tissues attached to the mandibles were removed and placed in 4% PFA overnight at 4°C and decalcified in 10% EDTA (pH 7.4) for about 4 weeks. The samples were dehydrated in ethanol at different concentrations of 50%, 70%, 80%, 90%, and 100%, each for 1 h. The samples were then mixed 1:1 with liquid paraffin, and the samples were placed in the mixture and placed in a 58°C incubator for 2 h. The samples were then placed in liquid paraffin and placed in a 58°C incubator for 2 h. The samples were then mixed 1:1 with liquid paraffin and placed in a 58°C incubator for 2 h. The samples were placed in a 58°C incubator for 2 h. Repeat 2 times. Paraffin embedding was performed and after the paraffin wax was completely hardened at room temperature, paraffin sections were prepared with a thickness of 9 μm and detailed labeling of section information. (*A*) The mandibles were isolated and the tissues are similar in size. (*B*) Paraffin slicing process.

**Figure. 10:**
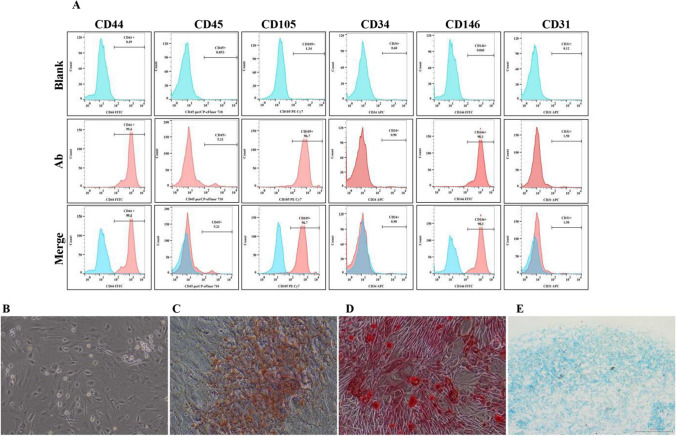
Cultivation and identification of mouse DPSCs. (*A*) The mesenchymal markers CD44, CD105, and CD146 and the hematopoietic markers CD45, CD34, and CD31 were detected by flow cytometry to identify the mouse DPSCs. (*B*) The cell was long spindle-shaped and grew in clusters, which were the typical morphology of mesenchymal stem cells in P3 generation of mouse DPSCs. (*C*) The adipogenic differentiation of P3 generation mouse DPSCs was obvious after 14 d of induction by oil Red O staining. (*D*) The osteogenic differentiation of P3 generation mouse DPSCs was significantly induced after 14 d induction by alizarin red staining. (*E*) The chondrogenic differentiation of P3 generation mouse DPSCs was significantly induced after 5 wk induction by Alsinland staining.

## Data Availability

The datasets generated during the current study are not publicly available, but are available from the corresponding author on reasonable request.

## Appendix

Figures 6, 7, 8, 9 and 10.
